# Trait-Mediated Variation in Seedling Performance in Costa Rican Successional Forests: Comparing Above-Ground, Below-Ground, and Allocation-Based Traits

**DOI:** 10.3390/plants13172378

**Published:** 2024-08-26

**Authors:** Nohemi Huanca-Nunez, Robin L. Chazdon, Sabrina E. Russo

**Affiliations:** 1 Yale Institute for Biospheric Studies, Yale University, New Haven, CT 06511-8934, USA; 2 Yale School of the Environment, Yale University, New Haven, CT 06511-2104, USA; 3 Department of Ecology and Evolutionary Biology, University of Connecticut, Storrs, CT 06269-3043, USA; 4 Forest Research Institute, University of the Sunshine Coast, 90 Sippy Downs Drive, Sippy Downs, QLD 4556, Australia; 5 School of Biological Sciences, University of Nebraska, Lincoln, NE 68588-0118, USA; 6 Center for Plant Science Innovation, University of Nebraska, Lincoln, NE 68588-0660, USA

**Keywords:** successional gradient, secondary forests, root traits, intraspecific, interspecific, growth, mortality

## Abstract

The interspecific relationship between functional traits and tree seedling performance can be inconsistent, potentially due to site-to-site or microsite variation in environmental conditions. Studies of seedling traits and performance often focus on above-ground traits, despite the importance of below-ground resource acquisition and biomass allocation to above versus below-ground functions. Here we investigate how varying environmental conditions across sites induce intraspecific variation in organ-level (above-ground, below-ground) and biomass allocation traits, affecting interspecific relationships between these traits and seedling performance. We analyzed trait expression for 12 organ-level and three allocation traits and their relationships with height growth (1716 seedlings) and mortality (15,862 seedlings) for 26 tree species across three sites along a forest successional gradient in Costa Rica. We found significant intraspecific differences across sites in all allocation traits, but only in three of seven above-ground and three of five below-ground organ-level traits. Allocation traits were better predictors of seedling performance than organ-level traits. Relationships between allocation traits and both growth and mortality varied among all sites, but for organ-level traits, only relationships with growth varied among sites. These results underscore that biomass allocation plays a key role in the earliest life stages of trees and that site-specific conditions can influence how functional traits mediate seedling establishment during succession.

## 1. Introduction

Multiple studies have linked plant species performance (i.e., growth and survival) with above-ground trait variation [1,2,3,4]. Studies on functional traits, such as specific leaf area [5], linked to photosynthetic potential [6], and wood-specific gravity [7], provide insights into how plant species allocate resources and are part of coordinated phenotypic variation leading to acquisitive vs. conservative strategies [1,4,8,9,10]. However, some studies have also highlighted weak to non-significant relationships between above-ground traits and plant performance [11,12,13].

One potential explanation for the weak explanatory power of functional traits is that within and among species, trait variation along environmental gradients obscures or weakens the general patterns of the interspecific relationship between above-ground functional traits and plant performance [11,14,15,16,17,18]. For example, in resource-rich environments, preferential investment in above-ground traits that enhance carbon gain supports faster growth rates. Conversely, in resource-poor environments, trait values leading to more conservative resource use reduce growth rates but enable stress tolerance [10,19,20]. Successional gradients are particularly suitable for understanding how changes in the environment, such as decreasing understory light availability along succession in a moist forest ecosystem [21,22,23,24], affect relationships between functional traits and plant performance. Moreover, the relative performance advantage of acquisitive versus conservative species is likely to vary along this successional gradient, since acquisitive species have phenotypes that are likely to better match earlier compared to later successional environments, particularly at the seedling stage [25]. These sources of variation can potentially affect relationships between traits, plant growth, and mortality.

Research on the relationships between functional trait variation and performance in tropical forests has largely focused on above-ground organ-level traits (leaf and stem traits) [15,26]. Meanwhile, other traits, such as organ-level below-ground traits, have received less attention, even though plant roots are responsible for acquiring nutrients and water from the soil necessary for above-ground function, plant growth, and survival. Yet, few studies examine how below-ground traits are related to the spectrum of acquisitive versus conservative strategies, as well as growth and survival [27,28,29]. Additionally, unlike organ-level traits, biomass allocation traits are thought to provide a more holistic view of the whole-plant capacity to distribute resources among roots, stems, and leaves [12,29,30,31]. Therefore, allocation traits are expected to align closely with a plant’s overall performance [1,18,32] and, at times, have been found to correlate more strongly with growth and mortality than above-ground organ-level traits [33]. Furthermore, understanding variation in biomass allocation could help us predict species’ responses to environmental changes, their role in ecosystems, and potential impacts on biomass yield. Nonetheless, the extent to which intraspecific trait and trait–performance relationships vary across sites remains largely unexplored, particularly for below-ground and allocation traits.

We investigated the hypothesis that variation in environmental conditions across different sites leads to intraspecific variation in seedling trait values, which may also affect the strength of the interspecific relationship between traits and seedling performance (growth and mortality), causing variation across sites. Data on 15 functional traits (Table S1) (above-, below-ground, and allocation traits) were collected from young seedlings of 26 woody species. We quantified the growth of 1716 young seedlings and the mortality of 15,862 established seedlings naturally growing in three forests in Costa Rica: a younger secondary forest (SEC1, 24 years), an older secondary forest (SEC2, 34 years), and a mature forest (MT). These sites differ in resource availability, with higher light availability in the secondary forests (SEC1, SEC2) than the mature forest (MT) [34]. While all these sites had similar soil carbon and nitrogen levels [35], they varied in soil phosphorus concentrations, a key limiting nutrient in many tropical soils [36], with SEC2 having higher phosphorus levels than MT and SEC1 [35].

Our study aimed to answer three questions: (1) How does the intraspecific functional trait expression of seedlings vary across sites? (2) Is there evidence of variation among sites in multivariate trait strategies? (3) How does interspecific trait variation influence seedling growth and mortality, and do these relationships differ across sites and different types of traits? We predicted that the expression of all traits would vary significantly across species and within species across sites. Due to differences in insolation, we predicted that intraspecific trait variation in seedlings across sites would produce a higher specific leaf area (SLA), resulting in a lower leaf mass fraction (LMF) but a higher root mass fraction (RMF) in the mature forest compared to the secondary forest sites. Owing to differences among sites in soil phosphorus, we also expected belowground traits and RMF to differ across sites, with greater investment in belowground resource acquisition capacity in the phosphorus-depleted sites (SEC1 and MT). Moreover, we predict that allocation traits would exhibit stronger relationships with seedling growth and mortality than organ-level traits and that both organ-level and allocation traits would exhibit site-specific differences in relation to seedling growth and mortality since both traits and performance rates are affected by resource availability. Additionally, since the interspecific growth-survival tradeoff suggests that investments in growth typically come at the expense of survival [1,4,37,38], we predicted that seedling growth and mortality would show opposing trends with organ-level and allocation traits (Table S2).

## 2. Methods

### 2.1. Study Site

The study was conducted at La Selva Research Station and surrounding areas in Sarapiquí province, Costa Rica. This region is classified as a tropical lowland wet forest [39]. Based on data collected over the past 30 years, the mean annual rainfall is ~4000 mm, with annual variation ranging from 3500 mm to 4500 mm, and there is no pronounced dry season. The mean annual temperature is ~26.5 °C [40]. Four 1-ha forest inventory sites (50 m × 200 m) were established in secondary successional forests in 1997, and another four 1-ha sites were established in 2005 (two in second-growth forests and two in mature forests) [41]. This study was conducted at three of these sites: an old-growth mature forest plot (MT) within La Selva Research Station (hereinafter, La Selva) and two second-growth forest plots, young mid-secondary (SEC1), locally known as Juan Enriquez, and older mid-secondary (SEC2), locally known as LSUR. These second-growth plots, one located outside and one within La Selva, have been undergoing natural regeneration for 24 and 34 years (in 2019), following pasture abandonment in 1995 and 1985, respectively. Plots were established for monitoring purposes in 2005 and 1997, respectively [42,43] (Table S3). The three study sites were selected to represent different stages of forest succession and are located relatively close to each other compared to other potential sites, facilitating comparison across them.

The three sites vary in resource availability, both above- and below-ground. At the time of this study, both secondary forests (SEC1, SEC2) had significantly higher understory light availability compared to the mature forest (MT), and the younger secondary forest (SEC1) had marginally higher light availability than the older secondary forest (SEC2) [34]. Despite similar soil carbon and nitrogen levels across the sites [35], they differ in soil concentrations of phosphorus, a critical limiting nutrient in many tropical ecosystems [36]. The older secondary site (SEC2) was found to have a higher concentration of soil phosphorus than the mature forest (MT) and the younger secondary site (SEC1) [35].

### 2.2. Seedling Monitoring: Mortality and Growth Data

We collected data on seedling mortality and growth separately but within the same sites. For mortality rates, we used data from a 6-year annual census (2005–2011), while the relative growth rate for height (RGRH) was monitored through a separate 26-month (2017–2019) experiment focused on younger seedlings. Specifically:

#### 2.2.1. Mortality Data

We employed a modified Gentry transect method [44] in each 1-hectare plot. This consisted of five parallel 2 × 100 m strips spaced 10 m apart, covering a total of 0.1 hectare per site. In these transects, all free-standing seedlings (>20 cm and <1 m in height) were tagged and identified by species. Annual censuses from 2005 to 2011 documented each seedling as alive, dead, or new. We calculated the mortality rate for each census interval as the percentage of stems that died from the census year indicated to the following census year within each of the five 0.02-hectare strips. Although some mortality events were captured during growth data monitoring (see below), the lower number of observations for the 26 targeted species made the comprehensive 6-year census data more adequate and essential for reliable mortality analysis.

#### 2.2.2. Growth Data

Twenty plots (1 m^2^) were established in each of three forest sites (60 plots total) in May 2017 to census young (<20 cm height) free-standing woody stems. The plots were randomly distributed, with a minimum distance of 2 m between each pair. In each plot, all free-standing seedlings were tagged, identified by species, and monitored every 60 days from June 2017 to September 2019. For each census, we recorded the seedling height to the nearest mm and documented whether each seedling was alive, dead, or new. All seedlings once recorded in the census were followed even after they grew to more than 20 cm. Subsequently, the relative seedling growth rate for height (RGRH), hereafter referred to as “growth”, was determined for each seedling divided by the number of months between two consecutive censuses.

### 2.3. Above-Ground, Below-Ground, and Allocation Trait Data

Above-ground, below-ground, and allocation traits were collected from young seedlings (20–50 cm in height) at the borders of each forest site in 2019. We collected data on 15 functional traits: specific leaf area (SLA), leaf dry matter content (LDMC), leaf nitrogen concentration (Leaf N%), leaf carbon concentration (Leaf C%), leaf thickness, leaf toughness, stem wood specific gravity (Stem WSG), specific root length (SRL), root tissue density (RTD), root nitrogen concentration (Root N%), root carbon concentration (Root C%), leaf mass fraction (LMF), stem mass fraction (SMF), and root mass fraction (RMF) (Table S1).

We measured traits (Table S1) on a total of 262 seedlings of 26 species, with four to six seedlings sampled per species at each site, representing a diverse range of taxonomic groups and light ecological strategies (Table S4). Our selection criteria targeted species that naturally occur in both old-growth mature and second-growth forests, encompassing eight light-demanding, ten shade-tolerant, and eight generalist species categories. The classification of these species was based on previous studies in the area [45]. This approach sometimes results in selecting species for second-growth forests that are less common in mature forests, and vice versa, so species do not necessarily represent the most abundant species from each site. We aimed to represent each species at a minimum of two different sites. At each site, 18 species were sampled. Among our study sites, two secondary forests; young mid-secondary (SEC1) and older mid-secondary sites (SEC2), shared 83% of the sampled species. Meanwhile, the young mid-secondary (SEC1) and mature forests (MTs), as well as the older mid-secondary (SEC2) and mature forest sites (MTs), shared 67% of the sampled species.

Images of scanned leaves were analyzed using ImageJ [46] to calculate each lamina’s area. We determined the fresh weight of these scanned leaves, and after oven-drying them for 48 h at 64 °C, we recorded their dry weight. We calculated specific leaf area (SLA) as fresh leaf area per leaf dry weight and leaf dry matter content (LDMC) as leaf dry weight per leaf fresh weight. Leaf thickness (mm) was measured at three points of the lamina for three leaves of each seedling, avoiding secondary veins whenever possible, using an absolute Digimatic Indicator ID-C series 543 (Mitutoyo, Kanagawa, Japan). Leaf toughness was measured at the same three points using a penetrometer (Chatillon by Ametek, Doral, FL, USA) with a constant tip size held in a plexiglass frame to ensure a consistent penetrometer angle for every measurement. Dried leaf tissue was ground to measure carbon (C) and nitrogen (N) concentrations through elemental combustion using a Costech Elemental Analyzer, Model 4010 (Costech Analytical Technologies, Valencia, CA, USA). We averaged the leaf-level measurements to calculate individual-level trait values.

Roots were cleaned carefully, scanned, and then analyzed with WinRhizo (version: Regular 2019; Regent Instruments, Quebec City, QC, Canada), allowing us to determine the total root length, mean root diameter, and total root volume. The roots were then oven-dried for 48 h at 64 °C to calculate their dry weight and ground to a uniform fine powder. Carbon (C) and nitrogen (N) concentrations were determined through elemental combustion. We calculated the specific root length (SRL) as the total root length over root dry mass and the root tissue density (RTD) as the root dry mass over fresh root volume. We then independently measured stem wood specific gravity (WSG) on portions of the main stem as the ratio of the oven-dry mass of the wood sample divided by the mass of water displaced by its green volume.

Leaf mass fraction (LMF) was calculated as leaf dry mass over total plant dry mass. Stem mass fraction (SMF) was determined as stem dry mass over total plant dry mass. Root mass fraction (RMF) was calculated as root dry mass over total plant dry mass.

### 2.4. Statistical Analysis

All analyses were performed using R statistical software 4.3 [47] (Table S5). To investigate intraspecific functional trait variation across sites (Question 1), we tested each of the 15 traits independently using seedling individual-level data. All variables were centered and scaled relative to their means and variances. We then used generalized linear models to analyze each trait. In these models, the specific trait was treated as the response variable, while the fixed explanatory variables included species and site (SEC1, SEC2, and MT) and their interactions to control for and test the variability within species across different sites. A normal error distribution was employed, and in instances where the data remained positively skewed even after transformation, a gamma distribution was utilized in the model. For all the models where significant intraspecific relationships across sites were found, we further examined them in sub models with each of the distinct light ecological strategies (light-demanding, shade-tolerant, and intermediate species).

To evaluate across-site variation in multivariate trait strategies (Question 2), we used principal component analysis (PCA) using the ‘prcomp’ function on species-level data by site. All variables were centered and scaled relative to their means and variances. We performed three separate PCAs: all organ-level above-ground traits, all organ-level below-ground traits, and all allocation traits. This resulted in three sets of species-level principal components reflecting variation in above-ground, below-ground, and allocation traits. The site difference was evaluated by a permutational multivariate analysis of variance [48], as implemented in the VEGAN package 2.6.4.

We assessed how above-ground, below-ground, and allocation traits influence the relative growth rate for height and mortality rates across sites (Question 3) using multivariate and univariate approaches, utilizing the functional trait value of each species collected at each specific site. Unlike other studies that use species mean trait values across all sites, we evaluated the relationships between site-specific species traits and seedling performance. We independently analyzed growth and mortality rates across each trait category: organ-level above-ground and below-ground, and allocation traits. For growth models with a normal error distribution, we used the lmer function in the LmerTest package 3.1.3. [49]. For mortality models with a zero-inflated beta error distribution, we employed the brms function in the brms package 2.21.0 in R [50]. We specifically tested how above-ground, below-ground, and allocation traits impact mortality and the relative growth rate for height using each trait category’s first and second principal components (PC1 and PC2) as a fixed explanatory variable. Accordingly, we fitted models that included either mortality rate or growth as the response variables, with fixed explanatory variables: (1) PC1 interacting with (2) sites (SEC1, SEC2, and MT) and (3) PC2 also in interaction with the site. We specifically tested independent models for above-ground, below-ground, and allocation traits. The growth models included seedling height and census time as fixed covariates to control for size-dependent and temporal variations, respectively, while the mortality models included census time but not seedling height as fixed covariates due to the unavailability of data. Additionally, we included species identity and plot as random terms.

We also used univariate analysis using site-specific species-level trait data to test how each trait (Table S1) interacted with the site to influence seedling growth and mortality. We included (1) each trait and (2) sites and their interactions as fixed explanatory variables. Seedling height and census time were also included as fixed covariates for growth and only census for mortality models. Additionally, we included species identity and plot as random terms, ensuring that species and spatial effects are properly controlled in our models. The goodness-of-fit of above- and below-ground and allocation traits were determined by computing marginal R^2^ (R^2^m) and conditional R^2^ (R^2^c) using the ‘r.squaredGLMM’ function in the ‘MuMIn’ package 1.47.5 [51] and the ‘r2_bayes’ function in the ‘performance’ package [52]. For the brms models, Markov Chain Monte Carlo (MCMC) sampling was performed on four chains, each with 20,000 iterations, discarding the first 10,000 iterations of each chain as burn-in. Convergence and diagnostics were judged for lmer models using the DHARMa package 0.4.6 [53] and for brms models visually when the MCMC chains were well mixed and when R-hat was ≤1.0 [50].

## 3. Results

### 3.1. Variation in Above-Ground, Below-Ground, and Allocation Traits among Species and within Species across Sites

All traits exhibited significant differences among species, but not all traits exhibited significant intraspecific variation among sites (Table 1). Specifically, among the seven organ-level above-ground traits analyzed, three showed intraspecific site differences (interaction between species and forest site), and among the five organ-level below-ground traits assessed, three displayed intraspecific site differences. Furthermore, among the traits that showed significant differences among sites, the proportion of species exhibiting intraspecific variation across a pair of sites was less than 36% for any given organ-level trait, except for leaf toughness, which showed intraspecific variability in 64% of the species (Table 1). In contrast, all three allocation traits displayed intraspecific trait variability between sites, with up to 58% of species showing significant differences between site pairs (Table 1). Specifically, 16 of the 26 species showed intraspecific variation in at least one trait between at least one pair of sites.

**Figure 1:**
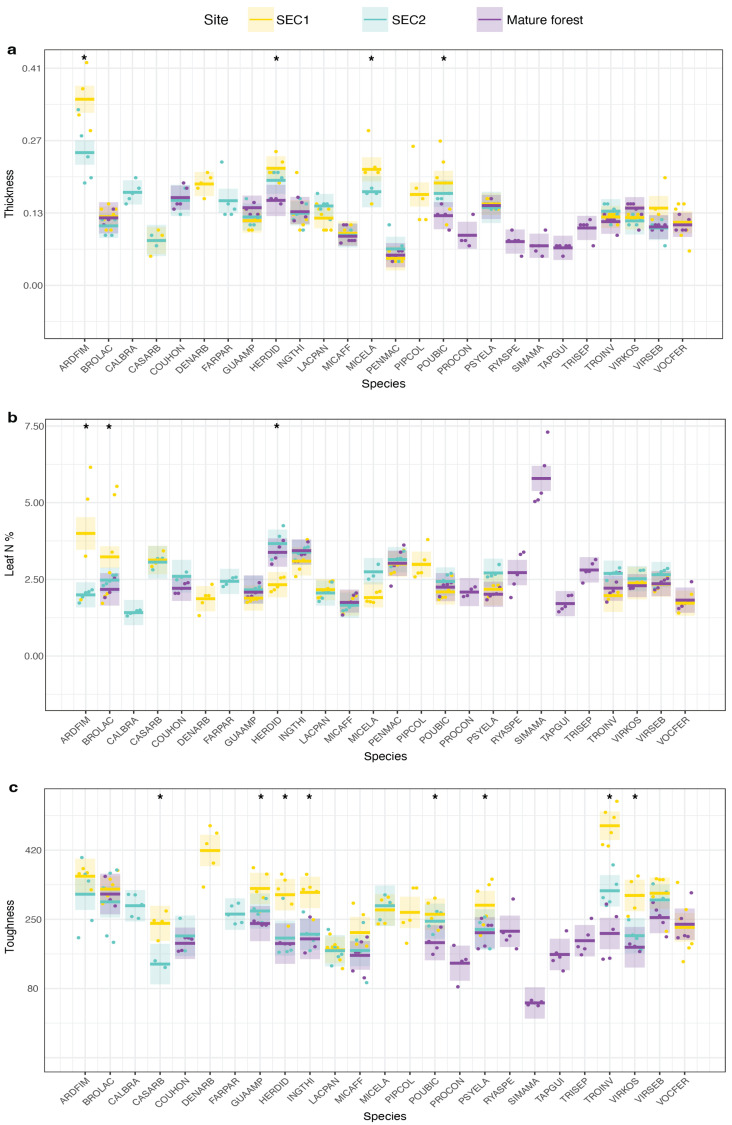
Above-ground species-trait variation of 26 tree species across a forest successional gradient in Costa Rica. Comparative representation of above-ground traits across species at three different sites. The shaded areas around the central lines in each boxplot represent the 95% confidence intervals, and the dots represent individual measurements for each seedling at each site. Asterisks (*) above the boxplots indicate significant differences among sites. (**a**) thickness, (**b**) Leaves N content, (**c**) Toughness.

**Figure 2:**
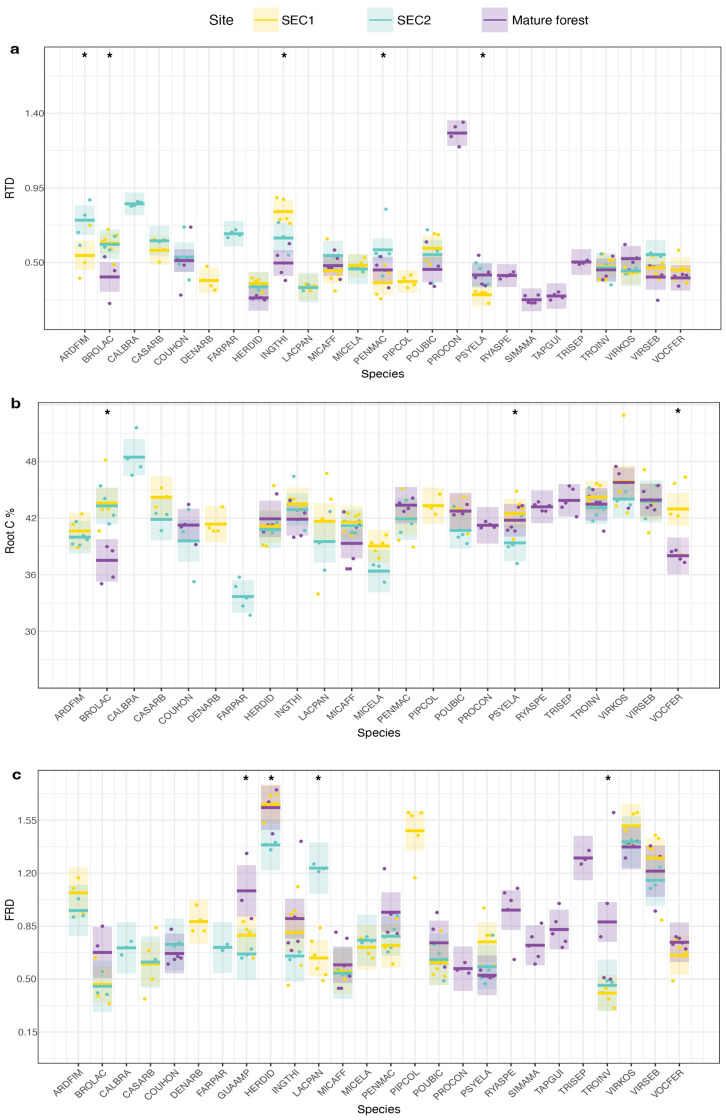
Below-ground species-trait variation of 26 tree species across a forest successional gradient in Costa Rica. Comparative representation of below-ground traits across various species at three different sites. The shaded areas around the central lines in each boxplot represent the 95% confidence intervals, and the dots represent individual measurements for each seedling at each site. Asterisks (*) above the boxplots indicate significant differences among sites. (**a**) Root Tissue density (RTD), (**b**) Root C content, (**c**) Fine root diameter.

**Figure 3:**
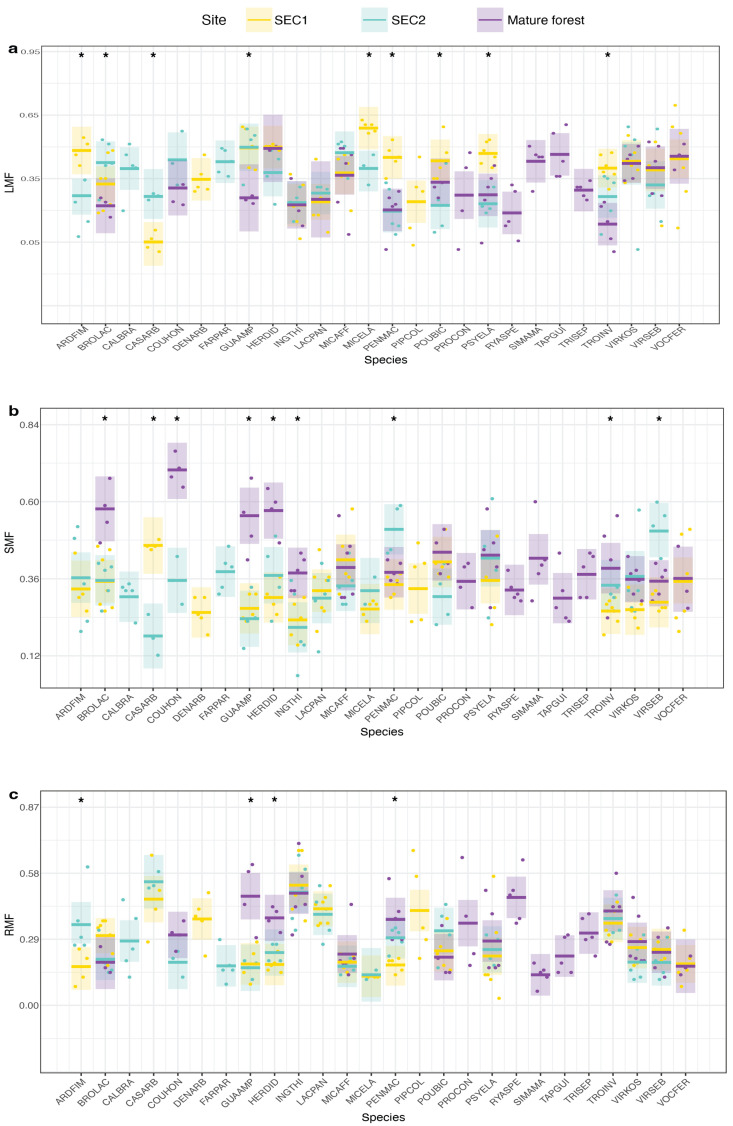
Allocation species-trait variation of 26 tree species across a forest successional gradient in Costa Rica. Comparative representation of below-ground traits across various species at three different sites. The shaded areas around the central lines in each boxplot represent the 95% confidence intervals, and the dots represent individual measurements for each seedling at each site. Asterisks (*) above the boxplots indicate significant differences among sites. (**a**) Leaf mass fraction (LMF), (**b**) Stem mass fraction (SMF), (**c**) Root mass fraction (LMF).

In summary, we found significant intraspecific site differences for the following leaf traits: leaf N% (F = 3.33, *p* < 0.01), thickness (F = 2.61, *p* < 0.01), and toughness (F = 3.76, *p* < 0.01) (Figure 1). We found intraspecific site differences for the following root traits: FRD (F = 3.57, *p* < 0.01), RTD (F = 3.52, *p* < 0.01), and root C% (F = 1.98, *p* < 0.01) (Figure 2). We found intraspecific site differences for all allocation traits: LMF (F = 2.80, *p* < 0.01), SMF (F = 4.64, *p* < 0.01), and RMF (F = 2.03, *p* < 0.01) (Figure 3). Moreover, whether species exhibited significant intraspecific differences across sites was generally not dependent on whether they were light-demanding or shade-tolerant: both groups showed significant intraspecific variation across sites for the reported traits. However, species with intermediate shade tolerance did not exhibit consistent intraspecific differences across sites (Table S6).

### 3.2. Variation in Multivariate Trait Strategies across Sites

**Figure 4:**
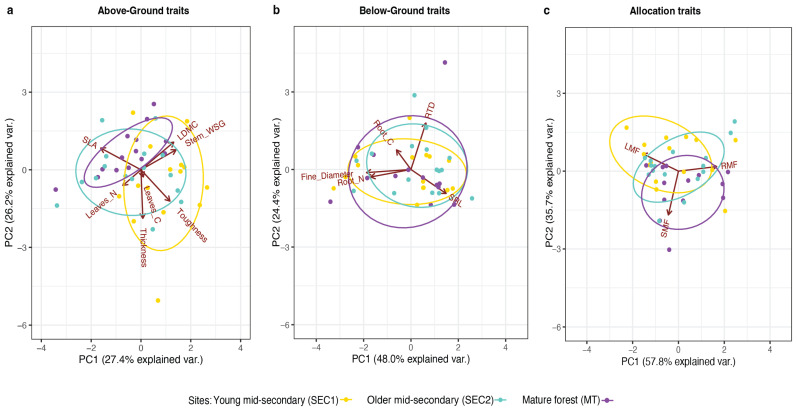
Principal component analysis of aboveground, belowground, and allocation traits of seedlings in Costa Rican successional forests. (**a**) Above-ground traits. (**b**) Below-ground traits. (**c**) Allocation traits. Color coding represents seedlings from different sites: mature show in purple, early-secondary in yellow, and mid-secondary in green. Trait abbreviations are explained in Table 1. Points are species’ mean trait values; ellipses are 95% confidence ellipses by site.

For the species selected in this study, PC1 accounted for a higher percentage of total variation in allocation and below-ground traits (57.8% and 48%, respectively), compared to above-ground traits (27.4%) (Figure 4). For above-ground traits (Figure 4a, Table S7), SLA was strongly negatively correlated with PC1, whereas stem WSG and LDMC were positively associated with PC1, corresponding to an interspecific acquisitive-conservative strategy spectrum. PC2 reflected a strategy spectrum of leaf defense traits, with the physical defense traits thickness and toughness loading negatively and the structural defense trait LDMC loading positively with PC2. For below-ground traits (Figure 4b, Table S7), SRL was positively associated with PC1, whereas FRD and root N were negatively associated with PC1, reflecting different organ-level strategies of resource absorption. SRL was negatively associated, and RTD and root C were positively associated with PC2, likely reflecting a spectrum of organ-level resource absorption versus tissue durability. For allocation traits (Figure 4c, Table S7), PC1 loaded strongly negatively for LMF and positively for RMF, reflecting a strategy spectrum of greater biomass investment in leaves versus roots, whereas PC2 loaded strongly negatively for SMF and positively for LMF, reflecting a strategy spectrum of greater investment in stems versus roots.

Multivariate trait strategies differed across sites, depending on the type of trait. Above-ground traits exhibited significant site-dependence (*p* < 0.01), biomass allocation traits exhibited marginally significant site-dependence (*p* = 0.08), but below-ground traits exhibited no significant site-dependence (Figure 4). Overall, for the species selected in this study, those at SEC1, the younger secondary forest with greater understory light availability, tended to build tougher leaves with lower SLA and higher LDMC, to have higher stem WSG, and to have greater biomass allocation to leaves and roots than stems. Species at SEC2, the older secondary forest with intermediate light availability and the highest soil phosphorus concentrations, tended to build leaves with higher SLA and leaf N and lower LDMC, to have lower stem WSG, and to have higher biomass allocation to leaves and roots than stems. Species at MF, the mature forest with the lowest light availability, tended to build thinner, less tough leaves with higher SLA, leaf N, and LDMC, to have higher stem WSG, and to have greater biomass allocation to stems and roots than leaves (Figure 4).

### 3.3. Relationships of Traits with Seedling Growth and Mortality

**Figure 5:**
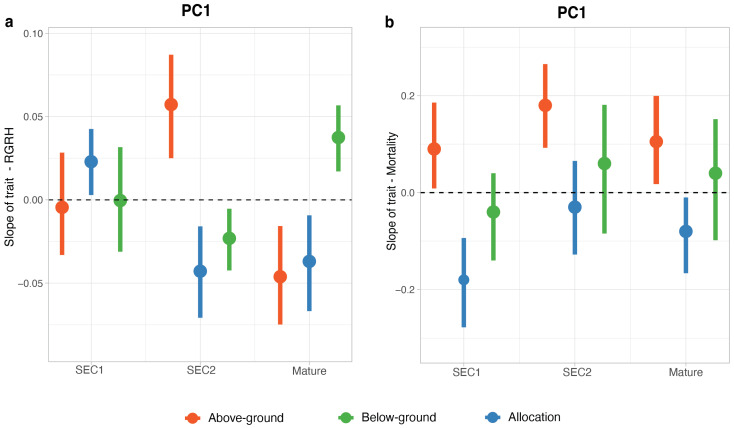
Variation in trait–growth and trait–mortality rate relationships across three forest sites. (**a**) presents the PC1 trait–RGRH (Relative growth rate for height—“growth”) relationship by site, and (**b**) presents the PC1 trait–mortality rate relationship by site. The points represent the estimated slopes from the model, and the error bars indicate the 95% confidence intervals. The dashed line at zero indicates no effect. In PC1, for above-ground traits, positive values indicate higher stem WSG, LDMC, and lower SLA, while negative values indicate the opposite. For below-ground traits, positive values indicate higher SRL and lower FRD, while negative values indicate the opposite. For allocation traits, positive values indicate higher RMF and lower LMF and SMF, and negative values indicate the opposite. Sites are abbreviated as young mid-secondary (SEC1), older mid-secondary (SEC2), and mature (MT) forests.

In multivariate analyses using PC1 and PC2 to represent multivariate trait strategies (Figure 4), the interspecific relationship of allocation traits with both growth and mortality varied significantly among sites, whereas these relationships exhibited less site-dependence for organ-level above and belowground traits (Figure 5). Specifically, for above-ground, below-ground, and allocation traits, a site-dependent response of the relative seedling growth rate for height (RGRH), hereafter growth, was observed for PC1 (F = 7.05, *p* < 0.01, F = 4.05, *p* = 0.03, F = 8.50, *p* < 0.01, respectively) (Figure 5a). However, PC2 did not show a significant site-dependent response to growth for above-ground and below-ground traits, but it did for allocation traits (F = 2.84, *p* = 0.05) (Figure S1a). In contrast, the relationships of above-ground and below-ground traits with seedling mortality were not markedly site-specific, except for allocation traits (Figure 5b, allocation: estimate = −0.18, 95% CI: −0.36 to −0.02). Although PC1 for above-ground traits was significantly related to mortality, this relationship did not vary significantly by site. Conversely, PC2 of above-ground traits and PC1 and PC2 of below-ground traits displayed neither site-dependent variation nor a significant association with mortality (Figure S1b). In contrast, both PC1 and PC2 of the allocation traits showed significant site-dependent relationships with mortality (Figure 5b and Figure S1).

Consequently, for the species selected in this study, different site-specific multivariate trait strategies were observed with respect to the interspecific relationships of traits with seedling performance. For above-ground traits, the positive slope observed in the older secondary site (SEC2) (Figure 5a) indicates that higher stem WSG, LDMC, and toughness and lower SLA (due to the positive correlation of WSG and LDMC and the negative correlation of SLA with PC1) corresponded to faster growth, whereas in the mature forest site (MT), the negative slope indicates that lower WSG, LDMC, and toughness but higher SLA corresponded with faster growth (Figure 5a). However, higher SLA and leaf N% corresponded to higher mortality across all sites (Figure 5b). For below-ground traits, in the SEC2 site, which had the highest soil *p* and intermediate light availability, the negative slope indicates that lower SRL and higher FRD and root N% (due to the positive correlation of SRL and the negative correlation of FRD and root N% with PC1) corresponded to faster growth, but in the MT site, higher SRL and lower FRD and root N% corresponded to faster growth (Figure 5a).

For allocation traits, the negative slope indicates that lower RMF (due to the positive correlation of RMF and the negative correlation of LMF and SMF with PC1) corresponded with faster growth at the SEC2 and MT forest sites, while at the younger secondary (SEC1) site, higher RMF correlated with faster growth (Figure 5a). However, higher LMF was associated with higher mortality at the SEC1 and MT forest sites (Figure 5b). However, there were also instances where no significant relationships were observed. For example, in SEC1, no significant relationship was found between above-ground or below-ground traits and growth. Regarding mortality, across all sites, below-ground traits did not significantly relate to mortality.

Furthermore, allocation traits had a slightly higher marginal R^2^ in predicting both growth and mortality, suggesting they have a slightly greater explanatory power compared to above-ground and below-ground traits. For growth models, the marginal R^2^ values were 0.10 [95% CI: 0.07, 0.13] for above-ground traits, 0.09 [95% CI: 0.06, 0.13] for below-ground traits, and 0.12 [95% CI: 0.09, 0.16] for allocation traits. For mortality models, the marginal R^2^ values were 0.16 [95% CI: 0.10, 0.22] for above-ground traits, 0.16 [95% CI: 0.09, 0.22] for below-ground traits, and 0.21 [95% CI: 0.17, 0.29] for allocation traits.

Based on univariate models, individual traits explained modest amounts of interspecific variation in both growth and mortality, even when including variation by site, indicating that considerable variation remains unexplained (Tables S7 and S8). However, allocation traits had a slightly higher marginal R^2^ in predicting both growth and mortality. The interspecific relationships of individual traits with growth and mortality also demonstrated site-dependence (Tables S8 and S9, Figure S2a–f). For growth, SLA was the strongest predictor among above-ground traits but exhibited a positive association with growth in MF, a negative association at SEC2, and no significant association at SEC1 (Figure S2a). FRD, root C, SRL, and RTD all had significant relationships with growth for at least one site (Figure S2b). For allocation traits, LMF was positively associated with growth at SEC2 and MF but negatively associated with growth at SEC1 (Figure S2b), whereas RMF was negatively associated with growth only at SEC2 and MF, and SMF was not associated with growth at any site (Figure S2c). For mortality, SLA and WSG were the strongest predictors among above-ground traits, showing a negative association across all sites for SLA and a positive association across all sites for WSG (Figure S2d). Only RTD had a significant relationship with mortality at SEC2 (Figure S2e). For allocation traits, LMF was positively associated with mortality at SEC2 and MF (Figure S2f), while RMF showed a negative association with mortality for all sites SEC1, SEC2 and MF (Figure S2f).

## 4. Discussions

Seedling establishment is a fundamental process during succession; thus, understanding the functional determinants of seedling growth and mortality is necessary for predicting forest regeneration trajectories [41]. Our study in Costa Rican forests focused on 26 species from 23 families (Table S4), including key tropical families like Moraceae, Rubiaceae, and Fabaceae, which are common in the region. We found that intraspecific functional trait variation (above-ground, below-ground, and biomass allocation) can be influenced by site-specific environmental conditions, such as soil nutrient availability and succession-associated variation in light, as found by [54]. However, intraspecific differences for the species selected in this study were generally not strongly associated with specific-species shade tolerance, suggesting that environmental factors can be just as influential on seedling phenotypes as species-level strategies like shade tolerance. While we cannot account for the possible effects of genotypic variation among sites, this finding is inconsistent with the prediction that more light-demanding species have greater plasticity [55]. As a result of intraspecific trait variation combined with differences in species composition, multivariate trait strategies of seedlings differed across sites and between secondary and mature forests, but less so for organ-level belowground traits than for organ-level aboveground and biomass allocation traits.

The relationships between functional traits and seedling growth and mortality also differed between sites, in part owing to site-related intraspecific trait variation. However, the strength of these relationships differed among traits and demographic rates. Relationships were strongest and exhibited the most site-related variation for allocation traits. In addition, allocation traits better predicted growth and mortality and more frequently exhibited significant relationships with these demographic rates than did organ-level traits, consistent with a study of seedlings in an Asian tropical forest [33]. These findings suggest that plasticity in biomass allocation is particularly critical to seedling establishment across spatially and temporally heterogeneous environments. In contrast, many organ-level traits did not vary significantly with seedling growth or mortality, although for growth there was often significant site-related variation. These findings suggest that functional traits may better predict variation in seedling growth than mortality. Our study highlights the key role of biomass allocation in the earliest life stages of trees, as well as the importance of environmental conditions, not just species-level shade tolerance, in shaping intraspecific trait variation and its relationships with demographic rates, which determine seedling establishment during tropical forest succession.

### 4.1. Above-Ground, Below-Ground, and Allocation Traits within Species Variation across Sites

We found intraspecific variation in six (leaf N%, thickness, toughness, FRD, RTD, and root C%) of the 12 organ-level traits and all three allocation traits (LMF, SMF, and RMF). Significant variation in leaf thickness and toughness is consistent with findings from [56], which reported intraspecific trait variation along an elevation gradient for six species across Puerto Rican mature tropical forests. On the other hand, while numerous studies have reported intraspecific variation in SLA in response to environmental factors [56,57,58], our study did not find significant intraspecific variation across the successional gradient in SLA for the species selected in this study. This was somewhat surprising as light availability is considered a key driver of variation in SLA [59], and our previous work found significantly lower light availability in mature forests compared to secondary forests [34]. Likewise, stem WSG did not show notable intraspecific variation. This lack of variation in stem WSG is consistent with findings from the Puerto Rican elevation gradient study [56], but inconsistent with significant intraspecific variation observed at later life stages [60]. Similarly, LDMC and leaf C%, root N%, and root C% did not exhibit intraspecific variation, although other studies have shown these traits to exhibit significant intraspecific variation at seedling and later tree life stages [61,62]. This limited variation we found could be related to constraints on tissue construction and stoichiometry [63,64] or that considerable variation in mechanical and structural resistance to physical damage to leaves can be achieved despite more restricted variation in commonly measured leaf traits like SLA [65]. Moreover, variation in these organ-level traits must be considered with respect to intraspecific variation in biomass allocation in a whole-plant context. For example, the reduced photosynthetic production per unit of leaf mass for leaves with high LDMC can be compensated for by greater allocation to leaf mass [54], which might limit intraspecific variation in LDMC, and we found significant intraspecific variation in LMF across sites. These results suggest that the magnitude of intraspecific trait variability depends on both the trait and the nature of the environmental gradient, as well as how functional integration [66,67] affects whole-plant performance in heterogeneous environments.

For below-ground traits, studies in grass, desert plant species, and temperate forests have shown that intraspecific variation can be influenced by elevation, soil properties, and species abundance [68,69,70]. However, in tropical forests, intraspecific variation remains an open question. We found variation across sites for SRL, FRD, and RTD. Consistent with a study in the deserts of northern Xinjiang, China, which reported intraspecific variation in RTD and SRL [69], our findings suggest adaptations to resource availability. In our study, RTD, SRL, and FRD appear to respond plastically to soil conditions. Variation in phosphorus levels, a key nutrient in many tropical soils [36], likely plays a significant role. The older secondary site (SEC2) has higher phosphorus concentrations [35], which may promote more efficient resource acquisition traits like higher SRL and FRD, while lower phosphorus sites may favor more conservative traits. These adaptations highlight the importance of soil nutrient availability in driving intraspecific trait variation in tropical seedling species.

For biomass allocation traits, we hypothesized that these traits would generally display greater intraspecific variation across sites, aligning with previous studies [71]. The median intraspecific variation in species across a pair of forest sites is 25% for allocation traits, compared to 17% for both aboveground and belowground traits. Specifically, within allocation traits, more species showed intraspecific variation in aboveground components (LMF and SMF) ranging from 18% to 58%, compared to the belowground component (RMF), which ranged from 7% to 25%.

### 4.2. Differences in Multivariate Trait Strategies between Sites

While allocation traits exhibited the highest intraspecific variation, particularly in LMF and RMF, for the species selected in this study, only the PCA strategies of above-ground traits varied significantly across sites. Multivariate trait variation across sites reproduces many trade-offs in function that have been previously observed. For example, the above-ground interspecific acquisitive–conservative strategy spectrum and strategy spectrum of leaf defense traits, the below-ground different organ-level strategies of resource absorption, and organ-level resource absorption versus tissue durability. Allocation strategy spectrum of greater biomass investment in leaves versus roots and strategy spectrum of greater investment in stems versus roots [32,72,73,74].

The significant site-dependence of above-ground traits underscores the adaptive responses of tropical seedlings to varying environmental conditions across the chronosequence. In SEC1, species appear to maximize structural support in high-light environments, as indicated by high LDMC and WSG, and generally lower SLA compared to SEC2 and mature forest (MF). In contrast, species at SEC2, with higher soil phosphorus concentrations and intermediate light availability, seem to prioritize efficient nutrient use, reflected by higher SLA, leaf N, lower LDMC, and lower stem WSG. These traits are expected to facilitate rapid growth and efficient resource use [36]. In mature forests (MFs), low light availability prompts species to allocate more biomass to stems, develop less tough leaves with higher SLA and leaf N, and increase stem WSG and LDMC, reflecting effects of both species turnover and intraspecific variation [29,54], aiming to maximize light capture and maintain structural integrity under shaded conditions. These traits are expected to enhance survival in shaded environments [19,20]. These patterns highlight the interplay between light availability, nutrient levels, and plant trait strategies in shaping tropical forest dynamics.

In contrast, below-ground and above-ground allocation strategies did not vary by site. However, in below-ground traits, PC1 loaded strongly positive for SRL and negative for FRD. Species with thinner roots and higher SRL are associated with a fast, resource-acquisitive strategy characterized by efficient resource foraging through increased root branching. Conversely, higher FRD is related to root longevity, indicating a resource-conservative strategy [28,75]. The unexpected alignment of higher FRD, a conservative trait, with higher Root N%, an acquisitive trait, is consistent with complex interactions between belowground traits and resource acquisition, in which the typical fast-slow leaf economics spectrum [10,74] may not fully apply to below-ground traits [28].

### 4.3. Trait-Performance Relationships and Site-Specific Variability in Seedling Growth and Mortality

Previous studies have noted that organ-level traits, though informative, often fall short in predicting overall seedling performance as they do not fully capture whole-plant dynamics [18,32,33]. These findings underline the significance of considering above- and below-ground biomass traits (LMF, SMF, and RFM), as biomass allocation is largely a zero-sum game, and thus allocation to one organ affects the others [30,31]. Our results show that allocation traits better predicted seedling performance than organ-level traits, emphasizing the crucial role of biomass allocation in early tree life stages. We also found that trait–performance relationships vary across sites for allocations of traits for both growth and mortality, whereas organ-level trait relationships sometimes vary significantly across sites for growth but never for mortality.

Consistent with traditional plant’s resource-use strategy for seedling performance, we observed that higher SLA was associated with faster growth rates at the mature forest (MF) site, aligning with our predictions [59,74]. Similarly, expected trends resulting from the growth-survival trade-off were evident at MF, where higher SLA was also associated with increased mortality [1,4,37]. However, some findings deviated from these expectations. At the older secondary site SEC2, with higher soil phosphorus concentrations and intermediate light availability compared to SEC1 and MF [34,35], lower SLA was associated with faster growth, in contrast to predictions from the leaf economics spectrum. Despite this, lower SLA and lower N% at SEC2 were still correlated with higher mortality. This suggests that at SEC2, resource availability supports faster growth despite traits typically associated with a conservative strategy, potentially due to higher soil phosphorus concentrations promoting efficient nutrient use. Thus, trade-offs in function depend on the environment, and investments in growth may not always come at the expense of mortality, given sufficient resource availability [76]. Given these observations, we recommend future studies of functional trait variation and its relationship to performance include a broader range of sites with varying resource availability.

For below-ground traits, expectations that faster-growing species exhibit higher SRL and lower FRD, advantageous for rapid soil resource uptake [77,78] were evident at the mature forest (MF). However, at SEC2, lower SRL and higher FRD were unexpectedly associated with faster growth, indicating a complex interaction between root traits and growth. This may be due to higher phosphorus levels at SEC2, enabling efficient resource acquisition despite conservative root traits, similar to the above-ground observations for SEC2. Additionally, below-ground traits had a non-significant influence on mortality, contrasting with [79], where below-ground traits were reported to influence seedling mortality across their 14 species studied in the dry tropical forests of Costa Rica. This discrepancy could be related to various differences between dry and moist tropical forests, such as light availability, which is not always lower in older than younger dry forests compared to moist forests.

Allocation traits, as predicted, showed that higher LMF and lower RMF were associated with increased mortality for all sites, but there was a contrast in biomass allocation strategies between SEC1 and the later successional stages (SEC2 and MT) and their effects on growth. At SEC2 and MT sites, higher LMF correlated with faster growth, aligning with hypotheses that higher LMF increases photosynthetic efficiency and growth in environments with a higher leaf area index [12,80,81]. In contrast, in the SEC1 site, where understory light availability was higher [34], faster growth was associated with lower LMF.

The differences between SEC2 and MT in the relationships of organ-level and biomass allocation traits with growth for the species selected in our study point to multifaceted growth strategies achieved by different combinations of functional trait expression [67]. At SEC2, conservative traits like lower SLA, higher LDMC, and higher FRD correlated with faster growth, indicating the value of tougher, denser leaves and roots. Yet, this forest exhibited faster growth with increased LMF, suggesting compensatory allocation to leaf mass that may help maintain whole-plant photosynthetic C-assimilation while also producing more physically robust leaves resistant to physical damage, a strategy that could simultaneously enhance both growth and survival for mid-successional species. In contrast, MF shows the expected pattern of acquisitive traits like higher SLA, lower LDMC, and lower FRD being linked with faster growth, and higher LMF complements this growth strategy. Moreover, SMF shifts from being positively associated with mortality in SEC1 to negatively associated with mortality in MT, indicating that as the canopy closes, stem allocation becomes crucial for accessing light, which supports findings by Zhang et al., 2024 [33], that identified stem-specific length as a strong predictor of growth and mortality in mature tropical forests. These results demonstrate the plasticity and complexity of plant growth strategies and the extent to which biomass allocation shifts in concert with changes in organ-level functional traits and the environment to influence growth and survival.

## 5. Conclusions

Seedling functional trait expression exhibited significant intraspecific variation for some of the studied species and traits. With the exception of leaf toughness, intraspecific trait variation was more pronounced in biomass allocation traits than in organ-level traits. Our findings highlight the significant influence of site-specific environmental conditions on the strength and direction of the relationships between functional traits and seedling performance. Additionally, they underscore the key role of biomass allocation in the earliest life stages of trees. Examining these dynamics in successional forests enhances our understanding of functional traits in seedling establishment and forest regeneration, informing regeneration and conservation practices. Future studies should explore how gradient differences in environmental variables, such as light and soil nutrients, impact trait-performance relationships.

## Figures and Tables

**Table 1:** Summary of Trait Differences Across Sites by Species. This table summarizes the results of multiple linear models examining the differences in various plant trait values across different sites. Additionally, the table displays the percentage of species that demonstrate significant differences in trait values (*p* < 0.05), adjusted using Tukey’s method for multiple comparisons across sites. Sites are abbreviated as young secondary (SEC1), older secondary (SEC2), and mature (MT) forests. The column “% Species with significant differences between pairs of sites” reveals the percentage of species in each pair of sites with significant trait differences, ranging from 0 to 100%. A dash (-) indicates 0% across any of the sites.

Abbreviation	Trait	Site by Species (F-Statistic, *p*-Value)	% Species with Significant Differences between Pairs of Sites
**Aboveground traits**			
LDMC	Leaf dry matter content (g/g)	0.80, 0.74	-
SLA	Specific leaf area (cm^2^/g)	1.06, 0.39	-
Leaf N%	Nitrogen concentration in leaves (%)	3.33, <0.01	SEC1-SEC2: 20%
			SEC1-MT: 17%
			SEC2-MT: 0%
Leaf C%	Carbon concentration in leaves (%)	1.25, 0.20	-
Thickness	Thickness (mm)	2.61, <0.01	SEC1-SEC2: 13%
			SEC1-MT: 17%
			SEC2-MT: 0%
Toughness	Toughness (N)	3.76, <0.01	SEC1-SEC2: 36%
			SEC1-MT: 64%
			SEC2-MT: 9%
Stem WSG	Specific gravity of stem wood (g/cm^−3^)	0.91, 0.59	-
**Belowground traits**			
SRL	Specific Root length (cm/mg^−1^)	1.13, 0.32	-
FRD	Fine Root Diameter (mm)	3.57, <0.01	SEC1-SEC2: 13%
			SEC1-MT: 17%
			SEC2-MT: 17%
RTD	Root tissue density (mg/cm^−3^)	3.52, <0.01	SEC1-SEC2: 22%
			SEC1-MT: 27%
			SEC2-MT: 18%
Root N%	Nitrogen concentration in fine roots (%)	1.33, 0.15	-
Root C%	Carbon concentration in fine roots (%)	1.98, 0.01	SEC1-SEC2: 7%
			SEC1-MT: 18%
			SEC2-MT: 9%
**Biomass allocation traits**			
LMF	Leaf mass fraction (g/g)	2.80, <0.01	SEC1-SEC2: 40%
			SEC1-MT: 33%
			SEC2-MT: 18%
SMF	Stem mass fraction (g/g)	4.64, <0.01	SEC1-SEC2: 20%
			SEC1-MT: 42%
			SEC2-MT: 58%
RMF	Root mass fraction (g/g)	2.03, <0.01	SEC1-SEC2: 7%
			SEC1-MT: 25%
			SEC2-MT: 8%

## Data Availability

The data is available in github (https://github.com/nohemihuanca/CR_Seedling_Trait_Variation accessed on 15 January 2024).

## Supplementary Materials

The following supporting information can be downloaded at: https://www.mdpi.com/article/10.3390/plants13172378/s1, Table S1. Above-ground and below-ground organ-level and biomass allocation traits measured for seedlings of 26 Costa Rican tree species. Table S2. Trait and their relationships with seedling performance (growth and mortality) Trait abbreviations: LDMC (leaf dry matter content), SLA (specific leaf area), Leaf N % (leaf nitrogen percentage), Leaf C % (leaf carbon percentage), Stem WSG (wood specific gravity), SLR (specific leaf area ratio), FRD (fine root density), RTD (root tissue density), Root N % (root nitrogen percentage), Root C % (root carbon percentage). Table S3. Stand characteristics of six 1-ha forest sites in successional and mature wet forests in Sarapiquí, Costa Rica. Names used in previous publications (e.g., Chazdon et al. 2010) are listed for comparison to previous studies at these sites. Sites are ordered by increasing successional age, from early successional (ES) to mature forest (MT). Table S4. List of codes, scientific names for all species in this study. Table S5. Model approach summary. Table S6. sub-models ANOVA results for site interaction across different light ecological strategies: LD, shade-tolerant (ST), and intermediate species (INT). The traits are grouped into three categories: above-ground organ-level, below-ground organ-level, and biomass allocation traits. Descriptions of trait abbreviations are in Table S1. These sub-models were analyzed separately to explore the interaction between site and species within each light group, as the main model did not include light ecological strategies groups as a factor. Table S7. Loadings of traits onto the principal components (PC1 and PC2). The traits are grouped into three categories: above-ground organ-level, below-ground organ-level, and biomass allocation traits. Descriptions of trait abbreviations are in Table S1. Table S8. Estimated variance explained for interspecific relationships of traits with relative growth rate in seedling height (RGRH) from univariate analyses. The traits are grouped into three categories: above-ground organ-level, below-ground organ-level, and allocation traits. Results are from a hierarchical model fit across seedlings with individual traits, site, and their interaction as fixed explanatory variables. Seedling height and census were also included as additional fixed variables. Species identity and plot were included as random terms. *R*^2^*m* (marginal R^2) represents the variance explained by the fixed factors alone, and *R*^2^*c* (conditional R^2) denotes the variance explained by both fixed and random factors. Descriptions of trait abbreviations are in Table S1. Table S9. Estimated Predictive Relationships of Traits to Mortality from univariate analyses. The traits are grouped into three categories: above-ground organ-level, below-ground organ-level, and allocation traits. Results are from a hierarchical model fit across seedlings with individual traits, site, and their interaction as fixed explanatory variables. Seedling census was also included as additional fixed variables. Species identity and plot were included as random terms. *R*^2^*m* (marginal R^2) represents the variance explained by the fixed factors alone, and *R*^2^*c* (conditional R^2) denotes the variance explained by both fixed and random factors. Descriptions of trait abbreviations are in Table S1. Figure S1. Variation of trait–growth and trait–mortality rate relationships across three forest sites. Panel A presents the PC2 trait–growth relationship, and Panel B highlights the PC2 trait–mortality rate relationship. Color coding represents the different used traits: above-ground traits in orange, below-ground traits in green, and allocation traits in blue. Sites are abbreviated as young mid-secondary (SEC1), older mid-secondary (SEC2), and mature (MT) forests. Figure S2. Above-ground, below-ground, and allocation traits relationship to growth and mortality rates across three forest sites. Panels a–c show slopes for independent RHGH by each functional trait for each site (colors with the 95% credible interval shaded) for above-ground, below-ground, and allocation trait. Panels d-f show slopes for mortality by functional trait for each site (colors with the 95% credible interval shaded) for above-ground, below-ground, and allocation trait. Color coding represents seedlings from different sites: mature shown in purple, early-secondary in yellow, and mid-secondary in green.
