# Supplementary material for: Trait-Mediated Variation in Seedling Performance in Costa Rican Successional Forests: Comparing Above-Ground, Below-Ground, and Allocation-Based Traits
Source: Plants (Basel). 2024 Aug 26;13(17):2378. doi: 10.3390/plants13172378 (PMC11397573; doi:10.3390/plants13172378)

Supporting information

Trait-mediated variation in seedling performance in Costa Rican successional forests: comparing above-ground, below-ground, and allocation-based traits

Nohemi Huanca-Nuñez, Robin L. Chazdon, Sabrina E. Russo

Table S1. Above-ground and below-ground organ-level and biomass allocation traits measured for seedlings of 26 Costa Rican tree species.

Abbreviation	Name	Units
**Aboveground organ-level traits**		
LDMC	Leaf dry matter content	g/g
SLA	Specific leaf area	cm^2/g
Leaf N %	Nitrogen concentration in leaves	%
Leaf C %	Carbon concentration in leaves	%
Thickness	Leaf thickness	mm
Toughness	Leaf toughness	g
Stem WSG	Specific gravity of stem wood	g/cm^3
**Belowground organ-level traits**		
SLR	Specific root length	cm/mg
FRD	Fine root diameter	mm
RTD	Fine root tissue density	mg/cm^3
Root N %	Nitrogen concentration in fine roots	%
Root C %	Carbon concentration in fine roots	%
**Biomass allocation**		
LMF	Leaf mass fraction	g/g
SMF	Stem mass fraction	g/g
RMF	Root mass fraction	g/g

Table S2. **Trait and their relationships with seedling performance (growth and mortality)** Trait abbreviations: LDMC (leaf dry matter content), SLA (specific leaf area), Leaf N % (leaf nitrogen percentage), Leaf C % (leaf carbon percentage), Stem WSG (wood specific gravity), SLR (specific leaf area ratio), FRD (fine root density), RTD (root tissue density), Root N % (root nitrogen percentage), Root C % (root carbon percentage).

Traits	Relationship with Seedling Growth	Relationship with Seedling Mortality	Source
LDMC	-	+	Pérez-Harguindeguy et al., 2013; Poorter, 2009; Weemstra et al., 2016; Wright et al., 2004
SLA	+	-	
Leaf N %	+	-	
Leaf C %	-	+	
Stem WSG	-	+	
SLR	+	-	
FRD	-	+	
RTD	-	+	
Root N %	+	-	
Root C %	-	+	

Table S3. **Stand characteristics of six 1-ha forest sites in successional and mature wet forests in Sarapiquí, Costa Rica**. Names used in previous publications (e.g., Chazdon et al. 2010) are listed for comparison to previous studies at these sites. Sites are ordered by increasing successional age, from early successional (ES) to mature forest (MT).

Site abbreviation used in this study	SEC1	SEC2	MF
Successional age in 2019 (y)	24	34	Mature Forest
Year of abandonment	1995	1985	NA
Site name in previous studies	Juan Enrique (JE)	Lindero Sur (LSUR)	El Peje primario (LEPP)
Location	Chilamate	La Selva	La Selva
Latitude	10.46°N	10.41°N	10.42°N
Longitude	84.07°W	84.03°W	84.04°W
Prior land use	Pasture	Pasture	Mature Forest
Surrounding landscape	Pasture, secondary, and mature forest	Mature and secondary forest	Mature forest

Table S4. List of codes, scientific names for all species in this study

**CODE**	**GENUS**	**SPECIES**	**FAMILY**
ARDFIM	*Ardisia*	*fimbrillifera*	Primulaceae
BROLAC	*Brosimum*	*lactescens*	Moraceae
CALBRA	*Calophyllum*	*brasiliense*	Calophyllaceae
CASARB	*Casearia*	*arborea*	Salicaceae
COUHON	*Coussarea*	*hondensis*	Rubiaceae
DENARB	*Dendropanax*	*arboreus*	Araliaceae
FARPAN	*Faramea*	*parvibractea*	Rubiaceae
GUAAMP	*Guatteria*	*amplifolia*	Annonaceae
HERDID	*Hernandia*	*didymantha*	Hernandiaceae
INGTHI	*Inga*	*thibaudiana*	Leguminosae
LACPAN	*Lacmellea*	*panamensis*	Apocynaceae
MICAFF	*Miconia*	*affinis*	Melastomataceae
MICELA	*Miconia*	*elata*	Melastomataceae
PENMAC	*Pentaclethra*	*macroloba*	Fabaceae
PIPCOL	*Piper*	*colonense*	Piperaceae
POUBIC	*Pourouma*	*bicolor*	Urticaceae
PROCON	*Protium*	*confusum*	Burseracea
PSYELA	*Psychotria*	*elata*	Rubiaceae
RYASPE	*Ryania*	*speciosa*	Salicaceae
SIMAMA	*Simarouba*	*amara*	Simaroubaceae
TAPGUI	*Tapirira*	*guianensis*	Anacardiaceae
TRISEP	*Trichilia*	*septentrionalis*	Meliaceae

TROINV	*Trophis*	*involucrata*	Moraceae
VIRKOS	*Virola*	*koschny*	Myristicaceae
VIRSEB	*Virola*	*sebifera*	Myristicaceae
VOCFER	*Vochysia*	*ferruginea*	Vochysiaceae

Table S5. Model approach summary.

Research Question	Model	Data	Approach	Variable response	Fixed effects	Random effects
How does intraspecific functional trait expression of seedlings vary across sites?	Model 1	Seedling individual-level data: 262 seedlings, 26 species, 3 sites.	Calculated independently for each trait, using a generalized linear model followed by pairwise Tukey-adjusted multiple comparisons.	Trait values	(1) Species in interaction with (2) site (categorized as SEC1, SEC2, MT)	NA
Is there evidence of variation among sites in multivariate trait strategies?	Model 2	Seedling species-level data by site: 26 species, 3 sites.	Principal Component Analysis (PCA) independently for above-ground, below-ground, and allocation traits, followed by permutational multivariate analysis of variance (PERMANOVA).	We used the following traits as columns in the PCA:<br><br>1. Above-ground traits: LDMC, SLA, leaves N %, leaves C %, leaf thickness, leaf toughness, stem wood specific gravity (WSG)<br>2. Below-ground traits: SRL, fine diameter, RTD, root N %, root C %<br>3. Allocation traits: RMF, LMF, SMF		
How does interspecific trait variation influence seedling growth and mortality, and do these relationships differ across sites and different types of traits?	Model 3a	For each category: above-, below-ground, and allocation. PCA axes values by site and species (From model 2). These values were then linked to a larger dataset with growth and mortality rates.	Multivariate approach: Linear models for growth and zero-inflated with beta error distribution for mortality models.	Growth (RGRH) and mortality rates	(1) PC1 interacting with (2) site (categorized as SEC1, SEC2, MT) and (3) PC2 also in interaction with the site (4) Seedling height (5) Census time	(1) Species identity (2) Plot
	Model 3b	Seedling species-level data by site: 26 species, 3 sites. These values were then linked to a larger dataset with growth and mortality rates.	Univariate Approach: Linear models for growth and zero-inflated with beta error distribution for mortality models. We run independent models for each of the 15 traits.	Growth (RGRH) and mortality rates	(1) An individual trait in interaction with (2) site (categorized as SEC1, SEC2, MT) (3) Seedling height (4) Census time	(1) Species identity (2) Plot

Table S6. sub-models ANOVA results for site interaction across different light ecological strategies: LD, shade-tolerant (ST), and intermediate species (INT). The traits are grouped into three categories: above-ground organ-level, below-ground organ-level, and biomass allocation traits. Descriptions of trait abbreviations are in Table S1. These sub-models were analyzed separately to explore the interaction between site and species within each light group, as the main model did not include light ecological strategies groups as a factor.

Trait	Group	Site (p-value)	Species (p-value)	Site:Species (p-value)
Thickness	INT	0.01	< 2e-16	0.45
Thickness	LD	0.00	0.00	0.01
Thickness	ST	0.00	< 2.2e-16	0.00
Leaf N %	INT	0.01	< 2.2e-16	0.12
Leaf N %	LD	0.00	< 2.2e-16	0.00
Leaf N %	ST	0.00	0.07	0.01
Toughness	INT	0.00	0.00	0.00
Toughness	LD	< 2.2e-16	0.00	0.03
Toughness	ST	0.00	0.00	0.11
FRD	INT	0.01	0.00	0.10
FRD	LD	0.00	< 2.2e-16	0.00
FRD	ST	0.05	< 2.2e-16	0.00
Root C %	INT	0.06	0.00	0.04
Root C %	LD	0.00	0.02	0.59
Root C %	ST	0.00	0.00	0.04
RTD	INT	0.00	0.00	0.00
RTD	LD	0.00	0.00	0.01
RTD	ST	0.00	< 2.2e-16	0.00
LMF	INT	0.00	0.00	0.01
LMF	LD	0.01	0.00	0.00
LMF	ST	0.81	0.00	0.10
SMF	INT	0.00	0.00	0.00
SMF	LD	0.00	0.03	0.00
SMF	ST	0.03	0.17	0.00
RMF	INT	0.01	0.00	0.30
RMF	LD	0.63	0.00	0.01
RMF	ST	0.08	0.00	0.04

Table S7. Loadings of traits onto the principal components (PC1 and PC2) . The traits are grouped into three categories: above-ground organ-level, below-ground organ-level, and biomass allocation traits. Descriptions of trait abbreviations are in Table S1.

PC1	PC2	Trait	Category
-0.57	0.29	SLA	above-ground
-0.29	-0.22	Leaf N %	above-ground
-0.02	-0.10	Leaf C %	above-ground
0.05	-0.66	Thickness	above-ground
0.41	-0.43	Toughness	above-ground
0.43	0.40	LDMC	above-ground
0.48	0.28	Stem WSG	above-ground
0.42	-0.46	SRL	below-ground
-0.51	-0.09	Root N %	below-ground
-0.56	-0.01	FRD	below-ground
0.42	0.11	Root WSG	below-ground
-0.17	0.36	Root C %	below-ground
0.20	0.80	RTD	below-ground
-0.67	0.37	LMF	allocation
-0.19	-0.93	SMF	allocation
0.72	0.10	RMF	allocation

Table S8. Estimated variance explained for interspecific relationships of traits with relative growth rate in seedling height (RGRH) from univariate analyses. The traits are grouped into three categories: above-ground organ-level, below-ground organ-level, and allocation traits. Results are from a hierarchical model fit across seedlings with individual traits, site, and their interaction as fixed explanatory variables. Seedling height and census were also included as additional fixed variables. Species identity and plot were included as random terms. R^2m (marginal R^2) represents the variance explained by the fixed factors alone, and R^2c (conditional R^2) denotes the variance explained by both fixed and random factors. Descriptions of trait abbreviations are in Table S1.

**Trait Category**	**Trait**	R^2m	R^2c
Above-ground	LDMC	0.081	0.107
Above-ground	SLA	0.090	0.115
Above-ground	Leaf N %	0.085	0.124
Above-ground	Leaf C %	0.088	0.115
Above-ground	Thickness	0.080	0.112
Above-ground	Toughness	0.090	0.140
Above-ground	Stem WSG	0.080	0.111
Below-ground	SLR	0.079	0.110
Below-ground	FRD	0.091	0.115
Below-ground	RTD	0.080	0.112
Below-ground	Root N %	0.079	0.108
Below-ground	Root C %	0.089	0.158
Below-ground	Root WSG	0.082	0.112
Allocation	LMF	0.092	0.148
Allocation	SMF	0.081	0.108
Allocation	RMF	0.090	0.114

Table S9. Estimated Predictive Relationships of Traits to Mortality from univariate analyses. The traits are grouped into three categories: above-ground organ-level, below-ground organ-level, and allocation traits. Results are from a hierarchical model fit across seedlings with individual traits, site, and their interaction as fixed explanatory variables. Seedling census was also included as additional fixed variables. Species identity and plot were included as random terms. R^2m (marginal R^2) represents the variance explained by the fixed factors alone, and R^2c (conditional R^2) denotes the variance explained by both fixed and random factors. Descriptions of trait abbreviations are in Table S1.

**Category**	**Trait**	**R-marginal**	**R-conditional**
Above-ground	LDMC	0.149	0.187
Above-ground	SLA	**0.180**	0.199
Above-ground	Leaf N %	0.169	0.190
Above-ground	Leaf C %	0.169	0.183
Above-ground	Thickness	0.162	0.194
Above-ground	Toughness	0.157	0.190
Above-ground	Stem WSG	0.148	0.195
Below-ground	SLR	0.157	0.190
Below-ground	FRD	0.153	0.190
Below-ground	RTD	0.149	0.187
Below-ground	Root N %	**0.165**	0.188
Below-ground	Root C %	0.154	0.186
Below-ground	Root WSG	0.149	0.192
Allocation	LMF	0.167	0.189
Allocation	SMF	0.162	0.192
Allocation	RMF	**0.182**	0.199

Figure S1. Variation of trait–growth and trait–mortality rate relationships across three forest sites. Panel A presents the PC2 trait–growth relationship, and Panel B highlights the PC2 trait–mortality rate relationship. Color coding represents the different used traits: above-ground traits in orange, below-ground traits in green, and allocation traits in blue. Sites are abbreviated as young mid-secondary (SEC1), older mid-secondary (SEC2), and mature (MT) forests.

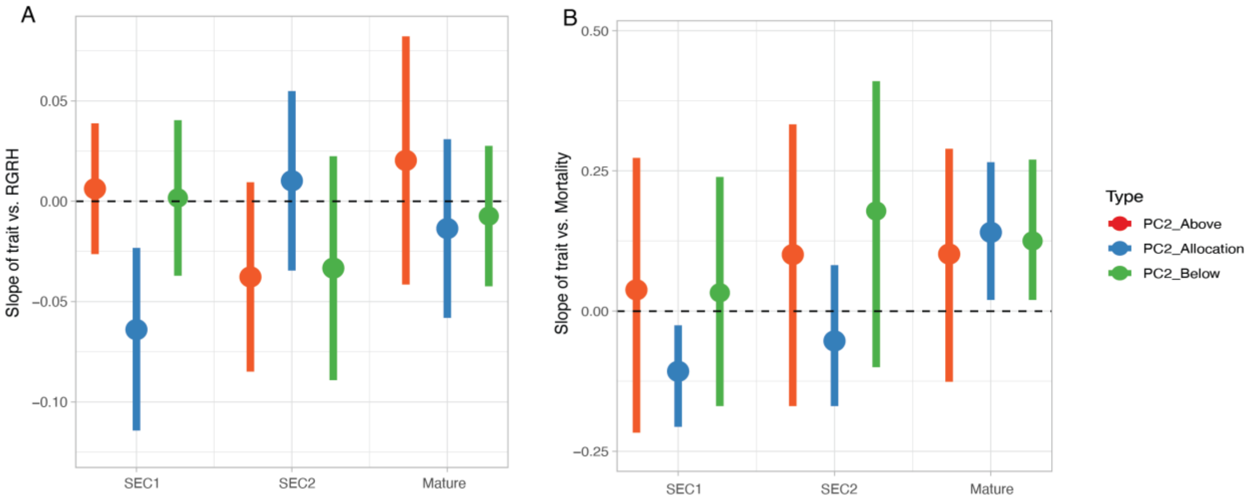

Figure S2. Above-ground, below-ground, and allocation traits relationship to growth and mortality rates across three forest sites. Panels a-c show slopes for independent RHGH by each functional trait for each site (colors with the 95% credible interval shaded) for above-ground, below-ground, and allocation trait. Panels d-f show slopes for mortality by functional trait for each site (colors with the 95% credible interval shaded) for above-ground, below-ground, and allocation trait. Color coding represents seedlings from different sites: mature shown in purple, early-secondary in yellow, and mid-secondary in green.

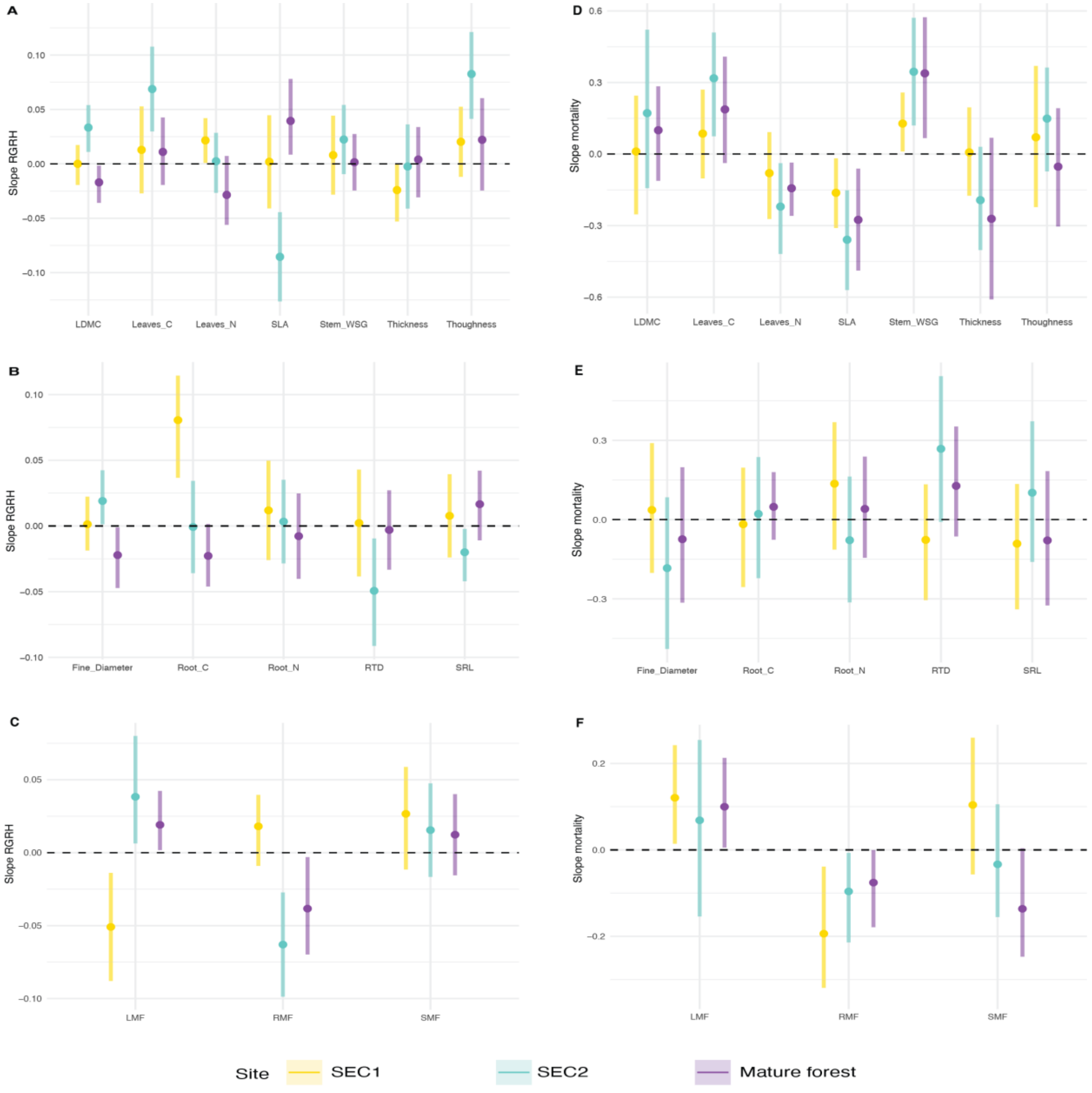

Supplement: Supplementary file 1 [file plants-13-02378-s001.zip › plants-3133092-supplementary.pdf]
